# Academic stress through salivary biomarkers: A multivariate exploration of cortisol, IL-1β, CRP, and IgA levels with sex-specific insights

**DOI:** 10.1371/journal.pone.0340316

**Published:** 2026-01-20

**Authors:** Rodrigo Castillo-Klagges, Camila Pezo-Sáez, Luis Aguila, Verónica Pantoja, Favián Treulen

**Affiliations:** 1 Escuela de Tecnología Médica, Facultad de Medicina y Ciencias de la Salud, Universidad Mayor, Temuco, Chile; 2 Centro de Excelencia de Biotecnología en Reproducción, Facultad de Ciencias Agropecuarias y Medioambiente, Universidad de La Frontera, Temuco, Chile; 3 Doctorado en Ciencias Morfológicas, Facultad de Medicina, Universidad de La Frontera, Temuco, Chile,; 4 Magíster en Neurociencias de la Educación, Escuela de Educación, Facultad de Ciencias Sociales y Artes, Universidad Mayor, Temuco, Chile; Padua University: Universita degli Studi di Padova, ITALY

## Abstract

Academic stress activates physiological responses mediated by the sympathetic-adrenal-medullary axis and the hypothalamic-pituitary-adrenal axis, leading to the release of biomarkers such as cortisol and proinflammatory cytokines. While stress physiology has been extensively studied in clinical populations, few studies have systematically examined the association between academic stress and multiple salivary biomarkers in undergraduates, particularly with attention to sex differences. This cross-sectional study investigated the relationship between self-reported academic stress survey measured via the SISCO inventory and four salivary biomarkers of cellular inflammation: cortisol, interleukin-1β, C-reactive protein, and immunoglobulin A in 81 undergraduates (53 females, 28 males). Biomarker levels were quantified using ELISA, and data were analyzed via multivariate approaches (ANOVA, Pearson correlations, and linear regression modeling). Participants were categorized as low (37%), moderate (35%), and high (28%) stress levels based on SISCO scores. Although no statistically significant associations were found between SISCO scores and individual biomarkers, multivariate analysis revealed a predictive model (R² = 0.14) combining all four biomarkers, with stress level predictions within ±20% of observed values. Males in the high-stress score showed lower cortisol trends but higher proinflammatory markers compared to females, suggesting divergent physiological stress responses by sex. These findings provide preliminary evidence for sex-differential association in self-reported academic stress with biological markers of inflammation, highlighting the potential of biomarker panels rather than single markers to capture the complexity of academic stress. In addition, this study establishes a methodological framework for combining psychometric tools with multi-biomarker analyses in stress research, addressing a critical gap in the literature on academic stress physiology.

## Introduction

The “stress response,” is essential for the survival of species, as it prepares the organism for dangerous situations [1,2]. This is an adaptive response that involves the activation of the sympathetic nervous system (SNS) and the hypothalamic-pituitary-adrenal (HPA) axis [3]. The activation of the SNS triggers fast short-term physiological modulations, increasing glycemia, blood pressure, heart rate, and stimulating the inflammatory and immune reaction [4–6]. This initial response is regulated by the HPA, that culminates in cortisol release, a glucocorticoid hormone with stress-regulating and anti-inflammatory functions [7]. This hormone modulates immune responses by inhibiting leukocyte proliferation and pro-inflammatory signaling [8–11].

Based on these physiological dynamics, stress response can be objectively assessed through the measurement of specific markers in biological fluids. For instance, blood analyses may reveal increased concentrations of pro-inflammatory cytokines (e.g., IL-1β) and acute phase proteins (e.g., C-reactive protein [CRP]). In addition, these inflammatory markers are also detected in saliva in response to psychological stress [12–15]. Similarly, salivary immunoglobulin A (IgA) may increase in response to acute stressors [6,16,17] but can be suppressed by prolonged exposure to stress [18]. Furthermore, the physiological effects of prolonged stress, associated with a systemic pro-inflammatory state [19] are considered a risk factor for various diseases such as cardiovascular disease, hypertension, diabetes, and cancer [20–24].

While these physiological manifestations reflect the biological impact of stress, it is important to consider that the origin and intensity of the stress response are largely shaped by cognitive and emotional processes. People are exposed to stressors daily, and humans can control what is perceived as stressful and how to respond [25,26]. Exaggerated or repeated negative interpretations, such as anxiety, and feelings of helplessness, represent catastrophic and maladaptive responses to stress that may prolong cortisol release [27,28]. This type of dysregulated response is particularly significant within the educational context. Academic stress is considered as the stress experienced by students because of recurring demands within academic environment. This phenomenon encompasses a complex, multidimensional interaction of psychological and environmental pressures that have been shown to exert significant and widespread effects on student’s psychological and physiological well-being [29–31]. Common academic stressors include academic milestones such as exams, tests, and oral presentations, which can elicit immediate physiological responses. Other stressors arise from persistent academic demands, sustained performance pressure, and continuous exposure to a challenging learning environment, all which can progressively impair mental and physical health [32–34]. External circumstances, such as an unfavorable family environment, may further amplify these effects [35]. It is known that stressful situations in students trigger psychological and physical symptoms such as anxiety, fatigue, insomnia, and signs associated with academic performance [36]. Although the detection of academic stress is particularly complex since psychological, social, and biological factors are involved [37]. Tools focusing on the stressor potential, symptoms and coping strategies induced by academic conditions have been developed, such as the Cognitive System Inventory for the Study of Academic Stress (SISCO) survey [38,39]. This self-reported academic stress survey has been employed in Latin America by several research groups [40–44].

Given the nature of academic stress, clarifying physiological correlations is essential to develop effective detection methods and preventive interventions. Thus, the objective of this study was to evaluate the association of salivary inflammation related biomarkers of cellular inflammation with academic stress levels in undergraduates, with attention to sex differences.

## Method

### Participants

The participant cohort were student volunteers from the Faculty of Medicine at Universidad Mayor, Temuco, Chile. A group of eighty-one students, aged between 18 and 30 years, was selected, including 53 women and 28 men. Sampling was non-probabilistic for convenience. The following exclusion criteria were considered to avoid directly or indirectly affecting the systemic inflammatory state [45–47]: pregnancy and/or breastfeeding, acute and/or chronic infections, chronic diseases, endocrine diseases, smoking, drug and/or alcohol abuse, and the use of any type of medication. Participants were invited to voluntarily participate by means of posters displayed at the university with a QR code, which redirected them to an online survey. This survey allowed the researchers to evaluate the inclusion/exclusion criteria of the participants and to collect personal information such as name, sex, age, academic program, academic year, telephone number and e-mail. The selected participants were contacted 3 days prior to sample collection and instructed to abstain from smoking, alcohol, and exercise. To avoid saliva contaminated with blood or other interferents, participants were instructed not to eat, drink, or brush their teeth for a period of two hours prior to sample collection.

### Study design

The participants were recruited via convenience sampling from October 30 to November 9 (Monday-Friday), 2023, between 9:30–11:00 AM. This period coincides with the conclusion of the academic semester. Participants were contacted individually and summoned to the university laboratory one day before sample collection. They received verbal and written instructions to abstain from food and drink (except water) for at least two hours before sampling. Participants were asked to sign a written informed consent form, which was reviewed and approved by the Scientific Ethical Committee of the Universidad Mayor (Resolution No. 0398). Then, the SISCO inventory was applied online. Participants were instructed to rinse their mouth with water, 10 minutes before sampling, and 1.5 ml of salivary sample was collected via passive drool into a 2 mL microcentrifuge tube. The samples collected were identified with the participant’s order number and the date of extraction. Immediately after collection, samples were centrifuged at 1000 x g for 10 minutes to remove cell debris, mucin, and debris. Subsequently, they were stored at −20ºC until analysis. Sample collection and analysis were performed in the university laboratory by the researchers.

### SISCO survey

Participants completed the SISCO-SV21 survey (S1 Survey), consisting of 21 questions that explored the three systemic-processual components of academic stress: stressors, symptoms and coping strategies. The first item, in dichotomous terms (yes-no), allows us to determine whether the respondent qualifies to answer the survey. The second is a single question item rated on a five-point Likert scale (1 = low; 5 = high), that measures the perceived intensity level of academic stress. The stressor section comprises seven items, Likert-type scale of six categorical values (0 = never; 5 = always), allow identifying the frequency in which the demands of the environment are valued as stressful stimuli, such as excessive workload, exam pressure, public presentations, inadequate classroom environments, or interpersonal conflicts are perceived as stressors. A representative item is: “How often do you get stressed by the overload of homework and schoolwork that I have to do every day?”. The symptoms and reactions section includes another seven items that assess the frequency of physical and psychological reactions to academic stress, including fatigue, irritability, headaches, and concentration difficulties. A representative item is: “How often do you experience reactions when you are stressed? Chronic fatigue (permanent tiredness)”. Finally, seven items allow to identify the frequency of use of coping strategies.

For scoring, the values obtained on the items related to stressors and symptoms, participants were classified into the following groups: low (<48%), moderate (>49% < 60%) and high academic stress level (61%><100%) according to the normative scale proposed by the original authors. The sections of this instrument can be used as a whole, separately or combined.

### Measures

The following biomarkers associated with cellular inflammation were analyzed: Cortisol (Cortisol ELISA Kit Abcam, Waltham, MA, United States), IL-1β (Human IL-1 beta ELISA Kit Abcam, Waltham, MA, United States), CRP (Human C Reactive Protein ELISA Kit Abcam, Waltham, MA, United States), IgA (Human IgA ELISA Kit Abcam, Waltham, MA, United States). Biomarker concentrations were reported as follows: cortisol, CRP, and IgA in ng/mL, and IL-1β in pg/mL. A microplate reader (BioTek Instruments EL800, Winooski, VT, United States) and its integrated analysis software (BioTek Gen5 software, Winooski, VT, United States) were used for reading results. The determinations of each biomarker were performed according to the manufacturer’s recommendations.

### Statistical analysis

Data normality and homogeneity of variance were assessed using Shapiro-Wilk and Levene’s test, respectively. Outliers were identified and removed to prevent distortion of the results, defined as values that deviated substantially from the distribution of the remaining data and detected using the interquartile range (IQR) criterion. To evaluate the relationship between variables, Spearman correlation coefficient was calculated to examine associations between biomarkers and academic stress percentage. Differences in biomarkers levels across stress categories (low, moderate, high) were analyzed using the Kruskal-Wallis test, followed by Dunn’s multiple comparisons test for post-hoc pairwise analysis. Linear regression models were applied to assess continuous associations between stress levels and biomarker concentrations. Additionally, comparisons of biomarker levels between men and women were performed using the Mann-Whitney U test. A p-value <0.05 was considered statistically significant. The results are expressed as mean ± SD.

Individual predictors were determined using analysis of variance (using the lm()-function in R, Vienna, Austria), and the coefficients of determination were used for numerical comparison of the variables. Finally, a multivariable model was constructed including all 4 variables.

## Results

The final sample included 81 undergraduate students, of whom 65.4% were women (n = 53) and 34.6% were men (n = 28). As shows Table 1, the mean academic stress percentage was similar between sexes (55% in women and 52% in men). The distribution by academic stage (seen as cycle/year of completion) was as follows from the first year until fifth year: 25.9%, 23.5%, 23.5%, 23.5%, and 3.6%.

**Table 1:** Distribution of participants across academic stress categories, mean academic stress scores and biological biomarkers associated with inflammation values by sex.

Sex	n	Academic stress categories - participants (No. and %)			Mean AS (%)	IL-1β (pg/mL)	CRP (ng/mL)	IgA (ng/mL)	Cortisol (ng/mL)
		*Low	*Moderate	*High					
Female	53	19 (36%)	17 (32%)	17 (32%)	55 ± 12	1.3 ± 2.4	0.6 ± 0.7	23 ± 18	5.2 ± 3.6
Male	28	11 (39%)	11 (39%)	6 (22%)	52 ± 11	1.9 ± 1.3	0.6 ± 0.8	39 ± 21	5.3 ± 3.6

Distribution of participants across academic stress categories (low, moderate, high), expressed as number and percentage of individuals within each sex, together with mean academic stress scores and mean salivary biomarker concentrations (IL-1β, CRP, IgA, cortisol; shown ± SD) by sex. Academic stress categories and mean academic stress were derived from SISCO-SV21 scores. Abbreviations are n: sample size; AS: academic stress; IL-1β: interleukin-1β; CRP: C-reactive protein; IgA: immunoglobulin A.

Of the total sample, 37% (30/81) were classified as low stress, 35% (28/81) as moderate stress, and 28% (23/81) as high stress. The mean percentage of academic stress within each group was 41% for the low-stress group, 55% for the moderate-stress group, and 68% for the high-stress group. The sex distribution within these groups revealed that women represented 63% (19/30) of the low stress group, 61% (17/28) of the moderate stress group, and 74% (17/23) of the high stress group, reflecting the sample composition.

**Fig 1:**
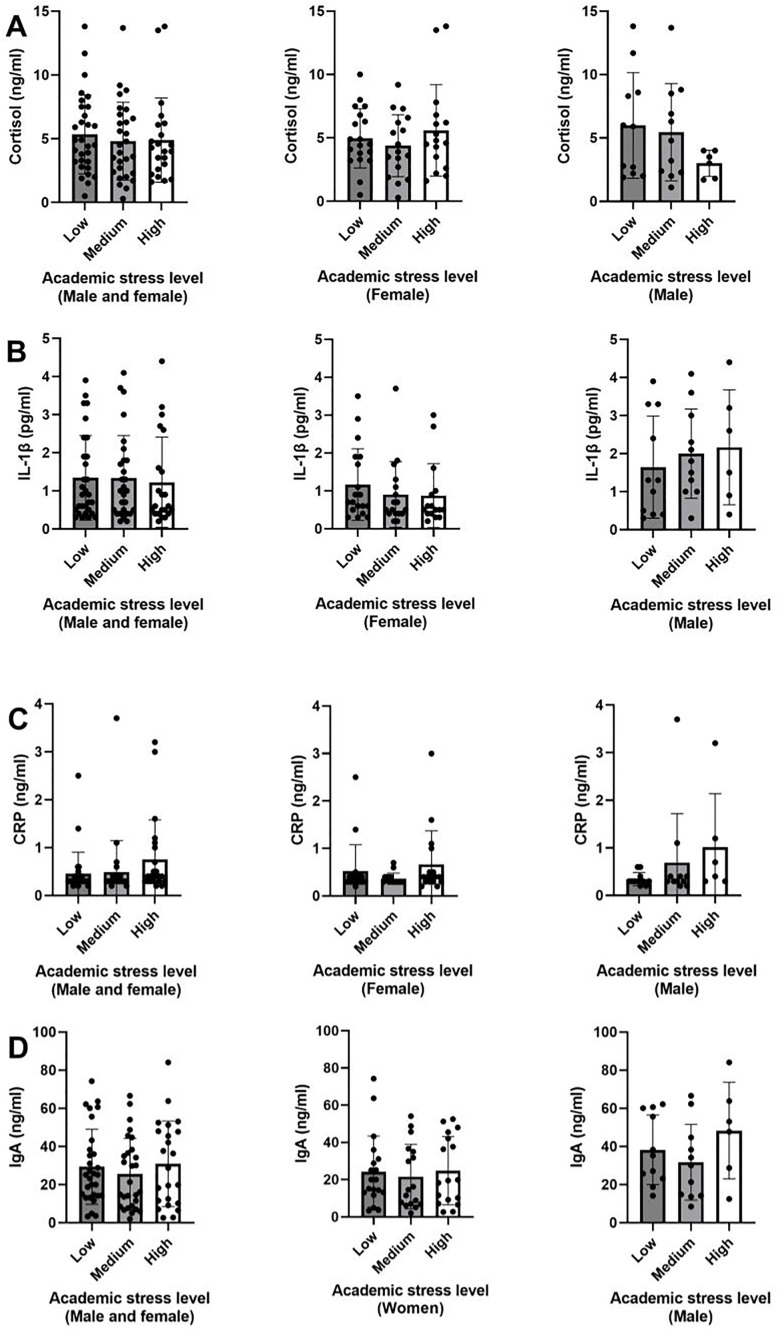
Salivary levels of cortisol, IL-1β, CRP, and IgA according to academic stress levels, analyzed globally and by sex. Bar plots illustrate the one-way ANOVA analysis. Each row corresponds to a different biomarker: (A) cortisol, (B) IL-1β (interleukin-1β), (C) CRP (C-reactive protein), and (D) IgA (immunoglobulin A). Within each row, the plots show the association between academic stress categories (low, moderate, and high), classified according to the SISCO questionnaire, and biomarker levels in the whole sample (left), women only (center), and men only (right). Results are expressed as mean ± SD.

When analyzing biomarker levels of inflammation (cortisol, IL-1β, CRP, and IgA) across academic stress categories (low, moderate, and high) (Fig 1), no statistically significant differences were detected between stress categories. However, in men, a descriptive trend was observed in which cortisol concentrations tended to decrease with higher levels of academic stress, while IL-1β and CRP tended to increase as stress scores increased.

**Fig 2:**
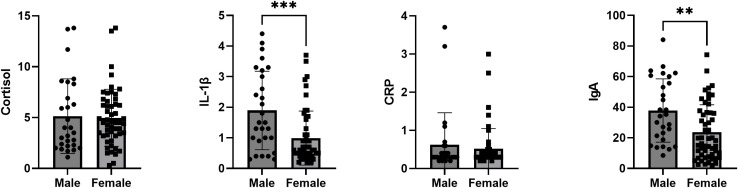
Comparison by sex of salivary levels of cortisol, IL-1β, CRP, and IgA. Bar plots represent the results of independent t-tests comparing mean salivary concentrations of cortisol (A), IL-1β (B), CRP (C), and IgA (D) between men and women. Results are expressed as mean ± SD. Statistical significance was set at *P* < 0.05. *** denotes *P* = 0.0002; ** denotes *P* = 0.0018.

To further examine sex-related differences, salivary biomarker concentrations were directly compared between men and women (Fig 2). Independent t-tests revealed that men presented higher mean levels of IL-1β and IgA compared to women (p = 0.0002 and p = 0.0018, respectively). No sex differences were observed for cortisol or CRP. These results indicate that sex-related variation exists in specific salivary biomarkers, regardless of the level of academic stress measured using the SISCO survey.

A simple linear regression analysis was applied to explore the relationship between salivary biomarkers and the percentage of academic stress (S1 Fig). There were no significant correlations for any of the biomarkers, either in the overall sample or when stratified by sex.

Finally, an exploratory multivariable model (S1 File) was developed to predict academic stress among male graduates of health-related degree programs using the combined biomarker measurements, as follows:


Stress level = 54.02 + 4.244 x CRP + 0.03758 x IGA − 0.7532 x CS − 1.24607 x IL−1β

CRP: C-reactive protein; IGA: immunoglobulin A; CS: cortisol; IL-1β: interleukin-1β.

The multivariable model had an R squared = 0.14 with predictions ranging approximately within +/- 20% of the stress levels.

## Discussion

In recent years, the use of salivary biomarkers to determine biological stress has been widely supported in the literature for its advantages over other types of biological samples [48–50], offering a noninvasive and accessible way to measure the physiological response to stress. This study aimed to identify correlations between biological markers associated with inflammatory processes detected in saliva with academic stress determined by the SISCO survey in undergraduate students of health-related degree programs. Despite extensive research on stress biomarkers, no study has simultaneously analyzed cortisol, IL-1β, CRP, and IgA in an academic stress context, nor explored sex-specific trends using multivariate modeling. Our work addressed this gap by combining psychometric assessments with a multi-biomarker approach.

Although, no significant correlations emerged between salivary biomarkers and stress levels; however, a trend was observed linking stress scores with cortisol, IL-1β, and CRP in male students. The absence of statistical significance may reflect interindividual variability in stress reactivity or limitations associated with sample size or convenience sampling. Nonetheless, these findings do not preclude the relevance of such biomarkers, as prior studies consistently show their modulation by acute and/or chronic psychological stress [51–54].

Salivary cortisol is a well-validated indicator of stress, and its concentration closely correlates with those in plasma [55]. Interestingly, our data showed that salivary cortisol tended to decrease in the high academic stress group. Although cortisol concentration increases in saliva after acute stress, in chronic periods or during stages of constant secretion, the cortisol levels decrease due to desensitization of the HPA axis and the presence of a proinflammatory state [54,56,57]. This phenomenon of HPA-axis depletion or glucocorticoid receptor resistance could explain the above state downward trend. Previous studies have reported greater cortisol reactivity in men than in women, with morning salivary cortisol identified as a predictor of academic stress in male undergraduates; conversely, women frequently report higher subjective stress, which may contribute to discrepancies between psychometric assessments and biological measures [58–60].

As cortisol, the inflammatory markers interleukin-1 beta and C-reactive protein have been used to assess inflammatory response to stress. In our study, both biomarkers showed an upward trend across academic stress categories, which may reflect the similar dynamics suggested by the concurrent decline in cortisol levels. IL-1β plays a central role in the stress-axis, acting not only as a sensitive biomarker but also as a mediator that modulates HPA-axis activity and autonomic responses. It is also described that men tend to show more pronounced inflammatory responses to stress compared to women, which is consistent with the higher levels of IL-1β in men obtained in our analysis. IL-1β sustained elevation has been linked to sleep disturbances, mood alterations, and impaired cognitive performance, aligning with symptomatology often reported in academic contexts [51,61–63]. Notably, experimental studies in chronically stressed animals demonstrated that IL-1β mRNA expression in submandibular glands is closely correlated with chronic stress exposure [64], supporting its role as a potential salivary biomarker of long-term stress load. Furthermore, another study demonstrated a significant increase in CRP concentration in response to a stressful task, suggesting an important role in dysregulation caused by stress [65]. However, further research is needed to determine whether there is a relationship between IL-1β and CRP concentrations with academic stress levels. This demonstrates that the measurement of academic stress is complex and cannot be categorized simply as acute or chronic stress.

Salivary immunoglobulin A is another potential biomarker of inflammatory processes, playing a crucial role in the immune system, particularly in mucosal immunity. Evidence regarding its relationship to psychological stress is conflicting. While several studies have reported transient increases in IgA following acute stressors, such as exams or oral presentations [66–68], others have documented decreases [7,69]. These divergent findings may reflect the dual role of IgA as an immediate defense mechanism and as a sensitive marker of sustained dysregulation of mucosal immunity. In our study, men showed significantly higher IgA concentrations than women, a result consistent with previous reports indicating that stress-induced immune responses may be more pronounced in men [54]. However, no direct correlation with academic stress was observed, suggesting that IgA reactivity may depend on situational and temporal factors that are not fully captured in a cross-sectional design. Future research should adopt longitudinal sampling strategies to better delineate whether IgA serves as a marker of acute stress reactivity, chronic stress suppression, or both, depending on the context.

In addition, we developed an exploratory multivariable academic stress predictor from proinflammatory biomarkers concentrations. The multivariable model responds in 14% of the change of academic stress. Other studies have established predictive models of post-traumatic stress in college students with correlations ranging from 19 to 38% [70,71]. Our results suggest that biomarker-based approaches may provide preliminary insights but are insufficient as stand-alone predictors. These findings should therefore be considered exploratory, underscoring the need for larger, longitudinal studies integrating psychological and contextual variables to improve predictive accuracy.

Finally, it is worth mentioning that unmeasured variables such as genetic predispositions, lifestyle habits (e.g., diet, sleep quality), or socioeconomic status could influence both stress perception and biomarker secretion. Importantly, another limitation concerns the lack of control for the menstrual cycle phase in female participants. Future studies should prioritize longitudinal designs, larger and more diverse sampling, considering the reproductive cycle phases, but also integrating undergraduates from a multidisciplinary cohort to validate the preliminary findings.

## Conclusions

In conclusion, although academic stress categories exhibited similar levels of the biological biomarkers associated with inflammation, we present the first multivariable model combining cortisol, IL-1β, CRP, and IgA to predict academic stress in undergraduate students of health-related degree programs. Although validation in larger cohorts is needed, this framework highlights the potential of biomarker panels rather than single markers to capture the complexity of academic stress physiology.

## Supporting information

S1 Fig Linear regression analyses between salivary biomarkers and academic stress percentage. The figure shows correlation and linear regression analyses between the percentage of academic stress, measured with the SISCO questionnaire, and salivary biomarker levels. Each row corresponds to a biomarker: (A) cortisol, (B) IL-1β, (C) CRP, and (D) IgA. Within each biomarker, results are displayed for the total sample (left), women only (center), and men only (right).
(TIF)

S1 Dataset Analyzed data. Classification of participants into academic stress categories (low, moderate, high) according to the SISCO questionnaire, measurements of salivary biomarkers (cortisol, IL-1β, CRP and IgA) and sex distribution (men and women).
(XLSX)

S1 Survey SISCO SV-21 Academic Stress Inventory (Spanish version). Self-administered questionnaire used to assess academic stress in university students. The instrument includes 23 items distributed across three dimensions: stressors, symptoms, and coping strategies (Barraza Macías, 2018).
(PDF)

S1 File Predictor composition of academic stress. This file contains the R code used to construct the academic stress predictor, detailing the variables included and the statistical approach applied.
(DOCX)
