# Supplementary figures and images for: Academic stress through salivary biomarkers: A multivariate exploration of cortisol, IL-1β, CRP, and IgA levels with sex-specific insights
Source: PLoS One. 2026 Jan 20;21(1):e0340316. doi: 10.1371/journal.pone.0340316 (PMC12818659; doi:10.1371/journal.pone.0340316)

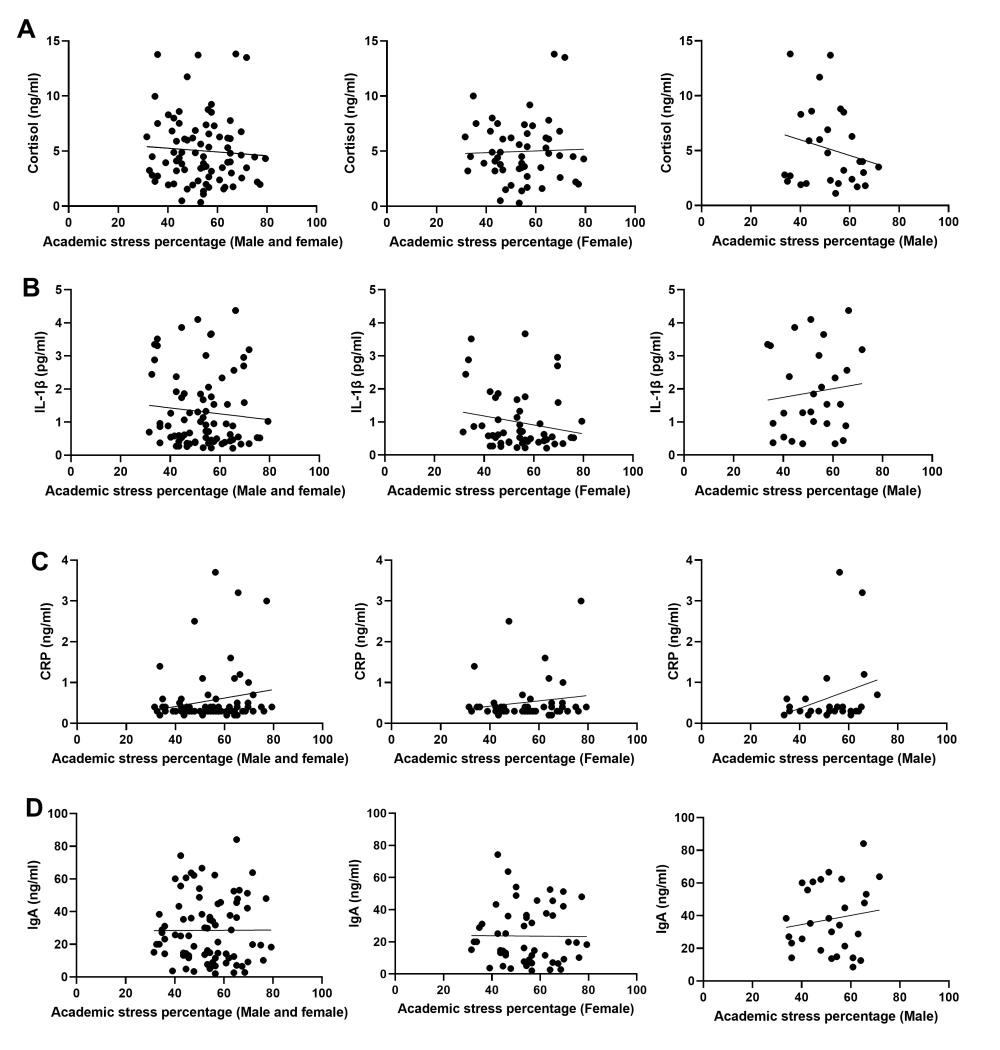

Supplement: S1 Fig — The figure shows correlation and linear regression analyses between the percentage of academic stress, measured with the SISCO questionnaire, and salivary biomarker levels. Each row corresponds to a biomarker: (A) cortisol, (B) IL-1β, (C) CRP, and (D) IgA. Within each biomarker, results are displayed for the total sample (left), women only (center), and men only (right). (TIF) [file pone.0340316.s001.tif]
