# Supplementary material for: Academic stress through salivary biomarkers: A multivariate exploration of cortisol, IL-1β, CRP, and IgA levels with sex-specific insights
Source: PLoS One. 2026 Jan 20;21(1):e0340316. doi: 10.1371/journal.pone.0340316 (PMC12818659; doi:10.1371/journal.pone.0340316)
Supplement: S1 Survey — Self-administered questionnaire used to assess academic stress in university students. The instrument includes 23 items distributed across three dimensions: stressors, symptoms, and coping strategies (Barraza Macías, 2018). (PDF) [file pone.0340316.s003.pdf]

SISCO INVENTORY SV-21

(Adapted from A. Barraza. Un Modelo Conceptual para el estudio del estrés académico. Revista Electrónica de Psicología Iztacala, 9 (2006), pp. 11-129)

1.- During this semester, have you had moments of worry or nervousness (stress)?

Yes

No

If you select "no," the questionnaire is complete. If you select "yes," proceed to question number two and continue with the rest of the questions.

2.- For greater accuracy, use a scale of 1 to 5 to indicate your stress level, where (1) is low and (5) is high.

**Never**	**Almost never**	**Rarely**	**Sometimes**	**Almost always**	**Always**
**N**	**AN**	**R**	**ST**	**AA**	**A**

How often does it stress you out:

**Stressors**	**N**	**AN**	**R**	**ST**	**AA**	**A**
The overload of schoolwork and assignments I have to do every day						
The personality and character of the teachers who teach my classes						
The way my teachers evaluate me (through essays, research papers, Internet searches, etc.)						
The level of demand from my teachers						
The type of work my teachers ask me to do (researching topics, worksheets, essays, concept maps, etc.)						
Having limited time to do the work assigned by my teachers						
The lack of clarity I have about what my teachers want						

4.- Symptoms (reactions) dimension

Instructions: Below is a list of reactions that, to a greater or lesser extent, tend to occur in some students when they are stressed. Answer by marking with an X how often each of these reactions occurs when you are stressed, using the same scale of values as in the previous section.

How often do you experience the following reactions when you are stressed:

Symptoms	N	AN	R	ST	AA	A
Chronic fatigue (permanent tiredness)						
Feelings of depression and sadness (downhearted)						
Anxiety, distress, or despair						
Concentration problems						
Feeling aggressive or more irritable						
Conflicts or tendency to argue or debate						
Lack of motivation to do schoolwork						

5.- Coping strategies dimension

Instructions: Below is a list of actions that, to a greater or lesser extent, some students tend to use to cope with stress. Respond by circling how often you use each of these actions to cope with stress, using the same scale of values as in the previous section.

How often do you use each of these actions to cope with your stress:

Strategies	N	AN	R	ST	AA	A
Focus on resolving the situation that concerns me						
Establish concrete solutions to resolve the situation that concerns me						
Analyze the pros and cons of the solutions designed to resolve the situation that concerns me						
Maintain control over my emotions so that I am not affected by what stresses me out						
Recall similar situations that have occurred in the past and think about how you resolved them						
Developing a plan to deal with what stresses me out and carrying out its tasks						
Focus on or try to find the positive aspects of the situation that is causing concern						

Coding: it is recommended to use the following numerical values for coding responses:

Never	Almost never	Rarely	Sometimes	Almost always	Always
**N**	**AN**	**R**	**ST**	**AA**	**A**
0	1	2	3	4	5

Quality control: to determine how valid the results of each questionnaire are, and therefore accept and integrate them into the database, more than 70% of the questions must be answered. In this regard, it is necessary that at least 16 of the 23 items in the questionnaire be answered; otherwise, that questionnaire will be invalidated.

Answer key: to interpret the results, it is necessary to obtain the overall average; the following steps are recommended:

- Only items from questions three, four, and five are taken for analysis.
- Each response on the scale is assigned the values already recommended for coding (see above).
- The mean is obtained for each item in each section, and then the overall mean is obtained.
- The mean is converted into a percentage, either using a simple rule of three or by multiplying the mean obtained by 20.
- Once the percentage has been obtained, it can be interpreted using two scales: one normative and the other indicative; the researcher decides which to use and reports it.

Normative scale focused on the population in which it was validated:

- From 0 to 48% mild stress level
- From 49% to 60% moderate stress level
- From 61% to 100% severe stress level

Indicative scale focused on the theoretical value of the variable:

- From 0 to 33% mild stress level
- From 34% to 66% moderate stress level
- From 67% to 100% severe stress level
