# Supplementary material for: Academic stress through salivary biomarkers: A multivariate exploration of cortisol, IL-1β, CRP, and IgA levels with sex-specific insights
Source: PLoS One. 2026 Jan 20;21(1):e0340316. doi: 10.1371/journal.pone.0340316 (PMC12818659; doi:10.1371/journal.pone.0340316)
Supplement: S1 File — This file contains the R code used to construct the academic stress predictor, detailing the variables included and the statistical approach applied. (DOCX) [file pone.0340316.s004.docx]

#creating the data frame

data <- structure(list(stress = c(33.7, 34.8, 35.9, 35.9, 40.2, 40.2, 42.4, 43.5, 44.6,

47.8, 47.8, 51.1, 51.1, 52.2, 52.2, 54.3, 55.4, 56.3, 57.6,

57.6, 60.9, 60.9, 63.0, 64.1, 65.2, 65.6, 66.3, 71.7),

CRP = c(0.2, 0.6, 0.4, 0.3, 0.3, 0.3 , 0.6, 0.2, 0.3, 0.3, 0.3, 0.2,

1.1, 0.3, 0.4, 0.3, 0.4, 3.7, 0.3, 0.4, 0.3, 0.2, 0.3, 0.3, 0.4, 3.2, 1.2, 0.7),

IGA = c(38.4, 27.1, 14.2, 23.2, 60.1, 25.8, 55.7, 35.3, 60.7, 62.2, 18.8, 38.4, 66.6,

30.1, 13.7, 14.9, 34.2, 62.4, 21.4, 44.8, 8.5, 14.2, 28.8, 12.5, 84.1, 47.8, 53.1, 63.9),

ILB = c(3.30, 3.30, 1.00, 0.40, 0.50, 1.30, 2.40, 0.40, 3.90, 1.30, 0.30, 1.30, 4.10, 1.80, 1.00,

3.00, 2.10, 3.60, 1.00, 1.50, 2.30, 0.30, 1.50, 0.40, 0.90, 2.60, 4.40, 3.20),

CS = c(2.8, 2.2, 2.7, 13.8, 8.3, 1.9, 2, 5.9, 8.6, 6, 11.7, 6.9, 4.8, 2.3, 13.7, 1.1, 2,

8.8, 8.5, 3.2, 6.3, 2.4, 1.7, 4, 4, 3, 1.8, 3.5)),

Names = c("stress_%", "CRP_mens", "IgA_mens", "ILbeta_mens"), row.names = c(NA, -28L),

class = c("tbl_df", "tbl", "data.frame"))

#See the data frame

View(data)

#Create the 5 estimators

est.CRP <- lm(stress ~ CRP, data=data)

est.IGA<- lm(stress ~ IGA, data=data)

est.ILB<- lm(stress ~ ILB, data=data)

est.CS <- lm(stress ~ CS, data=data)

est.4variables <- lm(stress ~ CRP + IGA + ILB + CS, data=data)

summary(est.CRP)

summary(est.IGA)

summary(est.ILB)

summary(est.CS)

summary(est.4variables)

confint(est.4variables)

#Normality of residuals

# CRP only ----------------------------------------------

residuosCRP <- residuals(est.CRP)

qqnorm(residuosCRP);qqline(residuosCRP)

shapiro.test(residuosCRP)

# IGA only ----------------------------------------------

residuosIGA <- residuals(est.IGA)

qqnorm(residuosIGA);qqline(residuosIGA)

shapiro.test(residuosIGA)

# ILBh only ----------------------------------------------

residuosILB <- residuals(est.ILB)

qqnorm(residuosILB);qqline(residuosILB)

shapiro.test(residuosILB)

# Cortisol only ------------------------------------------

residuosCS <- residuals(est.CS)

qqnorm(residuosCS);qqline(residuosCS)

shapiro.test(residuosCS)

# All four variables ------------------------------------

residuos4 <- residuals(est.4variables)

ajustados4 <- fitted.values(est.4variables)

qqnorm(residuos4);qqline(residuos4)

shapiro.test(residuos4)

hist(residuos4)

# Do predictions by replacing the specific values in examples

# Replace the values with the data from tests or just use the data already there.

# Highlight the line and press "Ctrl+Enter"

# PCR only ----------------------------------------------

(pred.CRP <- round(predict(est.CRP,newdata=data.frame(CRP=0.2),interval='prediction'),0))

# IGA only -----------------------------------------------------

(pred.IGA <- round(predict(est.IGA,newdata=data.frame(IGA=38),interval='prediction'),0))

# ILB only ----------------------------------------------------

(pred.ILB <- round(predict(est.ILB,newdata=data.frame(ILB=3),interval='prediction'),0))

# Cortisol only ---------------------------------------------------------

(pred.CSh <- round(predict(est.CS,newdata=data.frame(CS=3),interval='prediction'),0))

# All four variables --------------------------------------------------

(pred.4variables <- round(predict(est.4variables,newdata=data.frame(CS=3,CRP=0.6,IGA=27,ILB=3),interval='prediction'),0))

Stresslevel= 54.02 + 4.244 * 0.6 + 0.03758 * 27 - 0.7532 * 3 - 1.24607 * 3

print(Stresslevel)
